# Super-Repellent Paper Coated with Electrospun Biopolymers and Electrosprayed Silica of Interest in Food Packaging Applications

**DOI:** 10.3390/nano11123354

**Published:** 2021-12-10

**Authors:** Alvaro Lafraya, Cristina Prieto, Maria Pardo-Figuerez, Alberto Chiva, Jose M. Lagaron

**Affiliations:** 1 Novel Materials and Nanotechnology Group, Institute of Agrochemistry and Food Technology (IATA), Spanish Council for Scientific Research (CSIC), Calle Catedrático Agustín Escardino Benlloch 7, 46980 Paterna, Spain; alafraya@iata.csic.es (A.L.); cprieto@iata.csic.es (C.P.); mpardo@iata.csic.es (M.P.-F.); 2 Bioinicia R&D Department, Bioinicia S.L., Calle Algepser 65, nave 3, 46980 Paterna, Spain; achiva@bioinicia.com

**Keywords:** electrospinning, electrospraying, super-repellent coatings, PLA, PCL, PHBV, silica nanoparticles, easy emptying food packaging

## Abstract

In the current work, a super-repellent biopaper suitable for food contact applications was developed. To do this, three different kinds of biopolymers, namely polylactide (PLA), poly(ε-caprolactone) (PCL), poly(3-hydroxybutyrate-co-3-hydroxyvalerate) (PHBV), and hydrophobic silica microparticles (SiO_2_), were sequentially processed by electrohydrodynamic processing (EDHP). As a first step, the ultrathin biopolymer fibers were deposited onto a commercial food contact cellulose paper by electrospinning and, thereafter, the nanostructured silica was sequentially electrosprayed. The multilayer coated papers were annealed at different temperatures to promote adhesion between the layers and enhance the super-repellent properties. The developed coatings were characterized in terms of morphology, permeance to water vapor, adhesion, mechanical resistance, and contact and sliding angle. The resultant multilayer biopapers presented a hierarchical micro/nanostructured surface with an apparent water contact angle (WCA) higher than 155° and sliding angle (SA) lower than 10° for all the tested biopolymers used. Among the different multilayer approaches, it was observed that the paper/PHBV/SiO_2_ showed the best performance, in terms of water vapor permeance; resistance after the tape peeling-off test; and food super-repelling properties to water, yogurt, and custard. Overall, this study presents the successful generation of super-repellent biopapers coated with PLA, PCL, or PHBV along with hydrophobic silica microparticles and its effectiveness for easy emptying food packaging applications to reduce food waste.

## 1. Introduction

Cellulose paper is one of the most employed materials used in packaging along with glass, wood, and plastic. In the field of food packaging, paper-based materials, containing different non-renewable additives and coatings, are present in many of our daily food products, such as milk-based products, beverages, bakery products, etc., mainly all due to its advantages as substrate, such as low price, renewability, biodegradability, non-toxicity, lightweight, flexibility, and relatively good mechanical strength [1,2].

However, this type of material cannot be used alone for these and many other types of products due to its poor barrier properties and low sealability strength, as a result of its highly hydrophilic nature [2,3,4,5]. Frequently, paper-based products are often coated or laminated with additional materials, creating multilayer structures in an effort to enhance its mechanical, barrier, and sealing properties. What is often not known by the consumers is that these multilayer structures are frequently based on fossil fuel polymers which compromise its biodegradability, and ultimately contribute to the overall plastic waste problem [6]. Packaging made of petroleum-based materials represents a great environmental concern, since less than 2% are bioplastics and most of them end up in landfills [7]. Besides, the new Circular Economy Action Plan (nCEAP) recently issued by the European Commission (EC) shows that one of the key elements for the plastics industry is to develop alternative bio-based, as well as biodegradable and/or compostable plastics, as well as to identify which applications of these may be more beneficial to the environment [8]. Therefore, it is necessary to reduce the use of fossil fuel polymers as well as to find sustainable alternatives, not only for the structural packaging, but also for their coatings, adhesives, and overall intermediate layers. An interesting alternative could be the extension of the number of applications of cellulose paper by enhancing their technological properties [4], whilst preserving their biobased and biodegradable nature. In this regard, an increase in the hydrophobicity of the paper-based material by means of bio-based layers could be an appealing alternative for both the packaging industry and public research institutions worldwide, which are in continuous search for more sustainable and biodegradable alternatives [9,10].

With recent progresses on the development and fabrication of superhydrophobic surfaces for various applications, such as self-cleaning, anti-corrosion, anti-icing, friction reduction, etc. [11,12,13,14], the superhydrophobic coatings have attracted great attention over recent years [15]. Hydrophobic and superhydrophobic surfaces are often categorized based on their water contact angle (θ). Thus, a surface with θ > 90° is considered to be hydrophobic, while it is superhydrophobic when the apparent water contact angle (WCA or θ *) is >150° and the sliding angle (SA) is lower than 5°. These values cause the water droplets to instantaneously roll-off the substrate, creating a repellent effect [11,12,16]. Superhydrophobic cellulose paper presents a vast array of potential food applications, including microwavable food packages, fast food, and beverage containers [5], as this coating has several interesting properties, such as self-cleaning, water repellence, anti-frost, antibacterial, resistance against water and moisture, or extension of shelf life [1].

To obtain superhydrophobic cellulose paper, it is required to obtain a modified surface that should combine hierarchical micro-nano scale roughness together with low surface energy materials that confers superhydrophobic features to the paper [17,18]. Several techniques have been developed so far to produce superhydrophobic paper, such as plasma treatment [5], dip coating [19], spray coating [3], brush coating [18], chemical grafting [20], solution immersion [21], expansion of supercritical fluids [22], and electrospinning [4,23], among others. These methods can involve mono- or multilayer deposition and, depending on the technique, polymers and/or nanoparticles are employed. However, some of the above-mentioned techniques present some drawbacks, such as complexity, lack of versatility, or limited industrial applicability [11,12]. Alternatively, the use of electrohydrodynamic processing (EHDP) is a very simple and versatile technology that now can be easily integrated and scaled up into continuous production lines [11,24]. Electrospinning and electrospraying (both EHDP techniques) make use of high-voltage electric fields to produce fibers or particles with tailor-made physicochemical properties using a wide variety of viscoelastic polymer solutions. Electrospinning can either be used to generate sole membranes or, as a deposition technique, to coat layers on substrates of any type, such as polyethylene terephthalate (PET), low-density polyethylene (LDPE), steel, aluminum, thermoplastic corn starch (TPCS), paper, etc. [11,12,24,25,26,27].

The most employed materials to obtain superhydrophobic properties on paper are wax [17], resins, [14] fluorine-containing reagents [5,20,23,28], silane/siloxane/polydimethylsiloxane/potassium methyl siliconate [18,21,29], ethyl cellulose [4], carboxymethyl cellulose [13], and also nanoparticles (such as iron (III) chloride (FeCl_3_) [30], titanium dioxide (TiO_2_) [31], sepiolite and fatty acids [19], alkyl ketene dimer [22], zinc oxide (ZnO) [13], and hydrophobic silica (SiO_2_) [3,14,29,32,33]), or combinations thereof. However, the vast majority of studies have used materials that are not suitable for food contact applications and/or employed per- and polyfluorinated compounds, which can be toxic and imply a serious hazard for humans [4]. Only a few exceptions employed wax, ethyl cellulose or sepiolite/fatty acids [4,17,19] as appropriate food contact materials; however, to the best of our knowledge, there are no references using food grade hydrophobic silica. 

Superhydrophobic coatings have been widely used in many energy-related applications [34], but have not been so extended in the area of food packaging. To the best of our knowledge, research studies focused on generating superhydrophobic properties on cellulosic paper that are suitable for food contact applications are very scarce. Dias et al., for example, employed silica on paperboard in order to improve the barrier properties towards oxygen and water vapor; however, they did not aim at superhydrophobic performance [6]. The main objective of the present study was to explore the use of EHDP with three different types of biopolymers, namely polylactide (PLA), poly(ε-caprolactone) (PCL), and poly(3-hydroxybutyrate-co-3-hydroxy-valerate) (PHBV), along with hydrophobic food contact silica nanoparticles, to generate superhydrophobic coatings on cellulose paper. These biopolymers were selected since they are included on the European Union list of substances permitted for use in the manufacture of plastic materials and articles intended to come into contact with food described on Commission Regulation (EU) No 10/2011 [35]. All these biopolymers are either biodegradable in the environment or industrial compostable, while PLA and PHBV are also both fully bio-based (derived from natural and renewable resources) [7,36]. Regarding silica nanoparticles, the selected grade was, to the best of our knowledge, the only one commercially available that declares compliance to Regulation (EU) 10/2011, being suitable for food packaging applications. This is the first time that a super-repellent paper with improved barrier properties and suitable for food contact applications was developed employing EHDP. The super-repellent materials were characterized in terms of morphology, permeance to water vapor, mechanical resistance, adhesion, and hydrophobicity to water and food products.

## 2. Materials and Methods

### 2.1. Materials

A commercial food contact cellulose paper substrate was kindly provided by Grupo Lantero (Madrid, Spain). The supplier did not disclose any data on the paper formulation or its processing. The PLA used was Ingeo™ Biopolymer 3251D, supplied by Nature Works LLC (Minnetonka, MN, USA). According to the manufacturer, this biopolymer resin has a molecular weight (MW) of 5.5 × 10^4^ g/mol, and a polydispersity index (PI) of 1.62. PCL Capa™ 6800 was purchased from Perstorp UK Ltd. (Warrington, UK) with a number average molecular weight (Mn) of 80,000 g/mol. PHBV is a biowaste-derived copolyester produced by employing mixed microbial cultures fed with cheese whey as an industrial by-product of the dairy industry, and it was kindly provided by Universidade NOVA (Lisbon, Portugal). The composition of this PHBV copolymer was ~80 mol% PHB and ~20 mol% 3-hydroxyvalerate (HV). Prior to its use, it was purified with chloroform to produce a solid powder according to the a protocol previously reported [37]. Chloroform, trichloromethane (TCM), methanol (MeOH), dichloromethane (DCM), and 2,2,2-trifluoroethanol (TFE), at ≥99% purity for all of them, were purchased from Sigma Aldrich S.A. (Madrid, Spain). 1-butanol (BuOH) was purchased from Merck (Darmstadt, Germany). SIPERNAT D17 silica (SiO_2_), organomodified with dimethyldichlorosilane, with ≥97% purity, was obtained from EVONIK (Essen, Germany) and is certified as food contact grade by the manufacturer. The hydrophobic component is reported to bind to the surface hydroxyl groups present in the silica particles, generating the grafting process by dehydration to make the SiO_2_ more hydrophobic. Yogurt (Activia) was purchased from Danone (Paris, France), and custard, ketchup, and mayonnaise were purchased from Hacendado (Mercadona S.L., Tabernes Blanques, Spain).

### 2.2. Silica Particles Characterization

#### 2.2.1. Particle Size and Size Distribution

The particle size and distribution of the hydrophobic silica particles was carried out by employing a laser diffraction with the equipment Malvern Mastersizer MS2000 (Malvern Instruments, Ltd., Worcestershire, UK).

#### 2.2.2. Thermal Stability

The thermal stability of the silica particles was analyzed by thermogravimetric analysis (TGA) using a model Q-500 from TA instruments (New Castle, DE, USA). To this end, 12–16 mg of the powder sample was heated from 25 to 1000 °C at a heating rate of 10 °C/min under a nitrogen flow rate of 60 mL/min.

### 2.3. Fabrication of the Nanostructured Coatings

#### 2.3.1. Solution Preparation

The PLA solution for electrospinning was prepared by dissolving 12.5 wt % of the biopolymer in a DCM/TFE 5:5 (vol./vol.) solution. Similarly, the PCL solution was prepared by dissolving 10 wt % of the biopolymer in a chloroform/MeOH 8:2 (vol./vol.) solution. The PHBV solution was prepared by dissolving 2 wt % of the polymer in a chloroform/BuOH 7.5:2.5 (vol./vol.) solution. The three solutions were stirred at room temperature until complete dissolution. The silica dispersion was prepared by adding 0.75 wt % of silica in BuOH, before being stirred during 30 min at room temperature and then ultrasonicated for 3 min in an Ultraturrax T25 basic from IKA-Werke GmbH & Co. KG (Staufen, Germany).

#### 2.3.2. Electrospinning and Electrospraying

**Figure 1:**
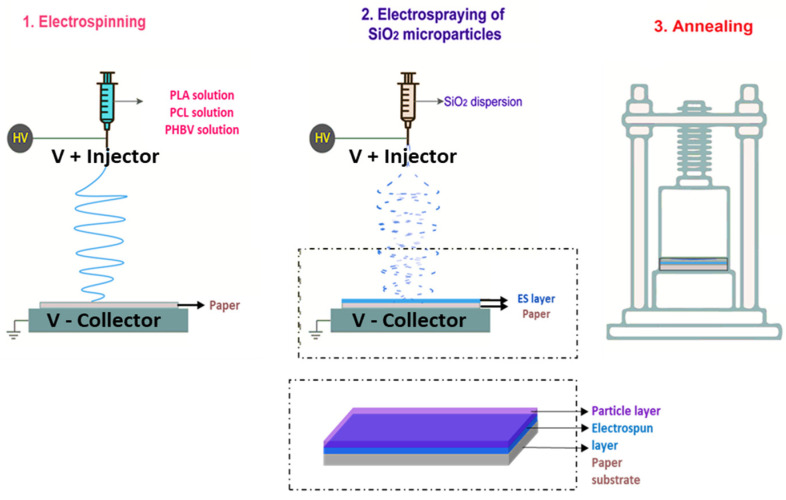
Schematic illustration of (**1**) deposition of the electrospun PLA, PCL, or PHBV fibers on cellulosic paper; (**2**) deposition of the electrosprayed silica (SiO_2_) microparticles on the electrospun layer; and (**3**) thermal annealing of the paper substrate/electrospun layer/SiO_2_ layer with a hot press without applying pressure.

Each solution or dispersion was first transferred to a 20 or 30 mL plastic syringe, which was connected through a polytetrafluoroethylene (PTFE or Teflon) tube to a stainless steel needle and sequentially electrospun/electrosprayed using a pilot-plant Fluidnatek™ LE-500 apparatus (Bioinicia S.L., Valencia, Spain, see Figure 1) working on roll-to-roll mode. The conditions employed for each process are summarized in Table 1 and the substrate was, in all cases, the commercial cellulose paper.

#### 2.3.3. Thermal Post-Treatment

The resultant multilayer structures of Paper/PLA/SiO_2_, Paper/PCL/SiO_2_, and Paper/PHBV/SiO_2_ were annealed in a hydraulic hot-press (model-4122 from Carver, Inc., Wabash, IN, USA) without applying pressure (both heat plates were at 4.5 cm distance from each other). To this end, the samples were placed on top of a Teflon sheet and different temperatures were evaluated for each one in order to find their optimal conditions for maximum hydrophobicity and layer adhesion. Table 2 gathers the different tested conditions.

### 2.4. Coatings Characterization

#### 2.4.1. Thickness

The coating thicknesses were measured using a digital micrometer Alfa Mirage Absolute series S00014 from Mitutoyo Corporation (Kawasaki, Japan), with ± 0.001 mm accuracy. The mean thicknesses were taken before and after the annealing treatment to characterize the different coated layers. Measurements were performed at six random positions, and the averaged values and the standard deviation were determined.

#### 2.4.2. Scanning Electron Microscopy

The morphologies of the hydrophobic silica and the resulting coatings were examined by field emission scanning electron microscope (FE-SEM) using a Hitachi S-4800 FE-SEM from Hitachi High Technologies Corp. (Tokyo, Japan). For cross-section visualization, the samples were cryo-fractured using liquid nitrogen. The materials were sputtered with a gold-palladium thin layer for 2 min under vacuum prior to analysis. An electron beam acceleration voltage of 5 kV and a working distance of 8–10 mm were applied. Image analysis was carried out using ImageJ 1.52a software (National Institutes of Health, Bethesda, MD, USA).

#### 2.4.3. Water Contact Angle Measurements 

Hydrophobic and superhydrophobic surfaces are defined based on the water contact angle (θ) value. A surface with θ > 90° is considered to be hydrophobic, while it is superhydrophobic when the apparent water contact angle (WCA or θ *) is >150°, and the contact angle hysteresis (CAH or Δθ *), the sliding angle (SA), and the shedding angle (SHA) are lower than 5° or 10° [16]. The typical example is the self-cleaning of nature’s lotus leaf, where the WCA is about 161° and the SA is only 2° [38].

The apparent water contact angle (θ) was measured at room temperature using a manual optical tensiometer in a Video-Based Contact Angle Meter Theta Lite TL 101 model from Biolin Scientific (Espoo, Finland) and the images were analyzed by means of the OneAttension software v 3.1 (Biolin Scientific, Espoo, Finland). A 5 µL drop of distilled water was deposited over the sample surface, and data were recorded 30 s after the droplet–surface contact. Measurements were carried out at room temperature conditions. For each sample, the average values were obtained by measuring 5 different positions. Contact angle determination employing liquid foodstuffs (yogurt, custard) were carried out under the same conditions as water, but the droplet volume was somewhat higher depending on the foodstuff used to assure reproducibility due to different rheological properties. Thus, yogurt droplets were of ca. 10 µL and custard of ca. 20 µL.

#### 2.4.4. Sliding Angle Measurements

Sliding angle determination was carried out on a 15 µL water droplet deposited over the surface, at room temperature, recorded with a video, while being tilted and analyzed with the ImageJ v 1.52a software. Sliding angle determination employing viscous foodstuffs, i.e., yogurt, custard, ketchup, and mayonnaise, was carried out with droplets of 20 µL for yogurt, 25 µL for custard, and 30 µL for ketchup and mayonnaise.

#### 2.4.5. Water Vapor Permeability Test

Water vapor permeability (WVP) tests of the coated paper samples were carried out with distilled water using the gravimetric method (ASTM E96-9) [39]. Briefly, 5 mL of distilled water were placed inside a Payne permeability cup with a diameter of 3.5 cm from Elcometer Sprl (Hermalle-sous-Argenteau, Belgium). The sample was placed in the cup, being exposed to the distilled water vapor on the coated side and secured with silicon rings under environmental conditions of 25 °C and 40% RH. The cups were weighted periodically using an analytical balance (±0.0001 g). Identical cups with aluminum foils were used as control samples to estimate the vapor loss through the sealing ring. The distilled water permeation rate was determined from the steady-state permeation slope obtained from a regression analysis of weight loss data per unit area vs. time, in which the weight loss was calculated as the total cup loss minus the loss through the sealing ring. The distilled water permeance was obtained by correcting the distilled water permeation rate for the permeant partial pressure. Three replicates per film sample were determined and averaged.

#### 2.4.6. Thermal Properties

Thermal analysis of the PLA, PCL, and PHBV biopolymers was carried out by differential scanning calorimetry (DSC) using a DSC 8000 analyzer from PerkinElmer, Inc. (Waltham, MA, USA). The thermal program was applied from 30 to 270 °C in a nitrogen atmosphere using a refrigerating cooling accessory (Intracooler 2, PerkinElmer, Inc.). The scanning rate was set at 10 °C/min in order to minimize the influence of this parameter on the thermal properties. An empty aluminum cup was used as the reference. Calibration was performed using an indium sample. The endothermic runs were carried out in at least triplicate.

#### 2.4.7. Tape Adhesion Test

A tape peeling test was carried out by employing Magic Scotch office tape (3M, Saint Paul, MN, USA). The adhesive tape was applied and pressed strongly by hand as a qualitative assay to determine the adhesive properties of the coating. Afterwards, adhesive tape was peeled off (speed ~2 mm/s) at an angle of 45°, as described in the literature [16,32].

#### 2.4.8. Statistical Analysis

The results were evaluated with the software STATGRAPHICS Centurion XVI version 16.1.03 (StatPoint Technologies, Inc., Warrenton, VA, USA). To identify significant differences among the samples, analysis of variance (ANOVA) was followed. Fisher’s least significant difference (LSD) was set at the 95% confidence level (*p* < 0.05). 

## 3. Results & Discussion

### 3.1. Silica Characterization 

**Figure 2:**
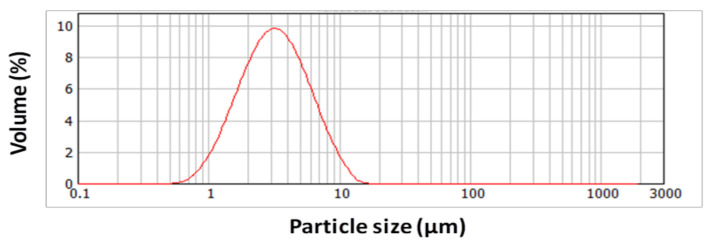
Particle size distribution of the as-received hydrophobic silica.

The average particle size (D50) of the commercial hydrophobic silica obtained by laser diffraction was 3.188 µm, presenting D10 and D90 values of 1.465 µm and 6.852 µm, respectively (see Figure 2). Other types of hydrophobic silica particles have been previously used on superhydrophobic coatings in other substrates in our research group, showing an average particle size of approximately 17 µm [12]. In that case, the resultant multilayer presented a hierarchical micro/nanostructured surface with an apparent contact angle of 157° and a sliding angle of 8° [12]. The size of the silica particles may influence the superhydrophobicity of the resultant coating, as occurred with PTFE particles reported by Morita et al., where an increase in the PTFE particle size greatly reduced the sliding angle without modifying the static contact angle [40].

**Figure 3:**
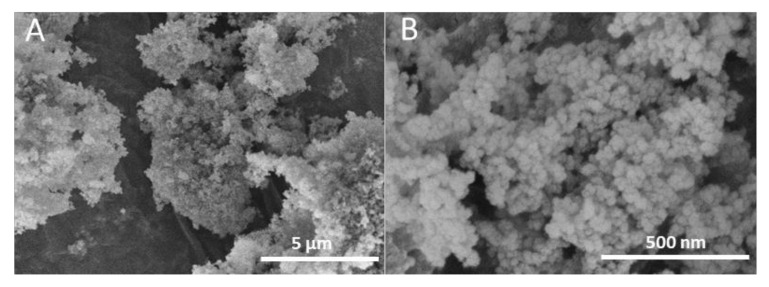
Field emission scanning electron microscope (FE-SEM) images of the as-received hydrophobic silica at two different magnifications (**A**,**B**).

On the other hand, the morphology of the hydrophobic silica microparticles was ascertained by FE-SEM. Figure 3 shows that silica formed clusters or agglomerates of several microns that were composed of fused nanoparticles of 40–80 nm. A similar morphology was recently reported for synthetic superhydrophobic self-cleaning NIR-reflective silica nanoparticles, obtaining a narrow size distribution of agglomerates made of monodispersed silica nanoparticles of around 40 nm [33].

**Figure 4:**
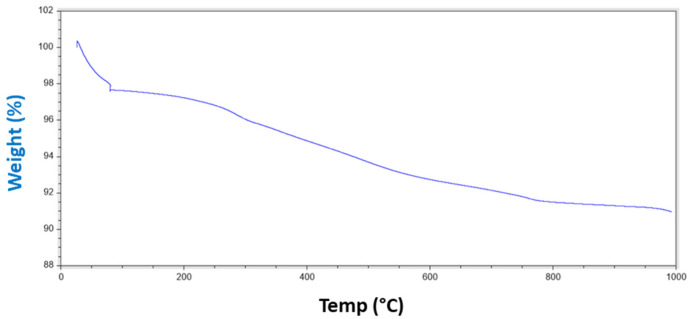
Thermogravimetric analysis (TGA) curve of the as-received hydrophobic silica.

The thermal stability of the hydrophobic silica microparticles was also analyzed by TGA under nitrogen atmosphere (see Figure 4). In the studied thermal range, the mass variation of the sample was low. TGA exhibited four main weight losses observed before. The first loss of mass was observed from room temperature to up to about 80 °C, and is attributed to free moisture. The weight loss between 80 and 150 °C was ascribed to the release of sorbed water molecules. The third mass loss, from 150 to 300 °C, was assigned to the decomposition of organic groups and loosely bound Si–(OR) groups. Finally, from 400 °C onwards, the organosilicate framework was thermally degraded. It can also be observed that at least 91% of the mass remained stable at 1000 °C, in accordance with results reported previously in which different hydrophobic silicas were analyzed [41,42].

### 3.2. Characterization of Electrospun Biopolymers and Electrosprayed Silica 

The aim of this study was to generate thin coatings since multilayer structures may comprise coatings ranging from 30 to 150 µm [43]. In this case, the aim was to develop a superhydrophobic effect as well as improved barrier properties for each paper/biopolymer system, employing the least amount of material. Thus, the different thicknesses of the uncoated paper as well as the samples produced by electrospinning using PLA, PCL, and PHBV, as well as subsequent electrospraying with the hydrophobic silica microparticles, were measured and gathered in Table 3. The uncoated paper presented a thickness of 77.9 ± 2.0 µm, while the coatings of PLA, PCL, and PHBV electrospun mats deposited over paper were approximately 20.6, 34.6, and 14.3 µm, respectively.

**Figure 5:**
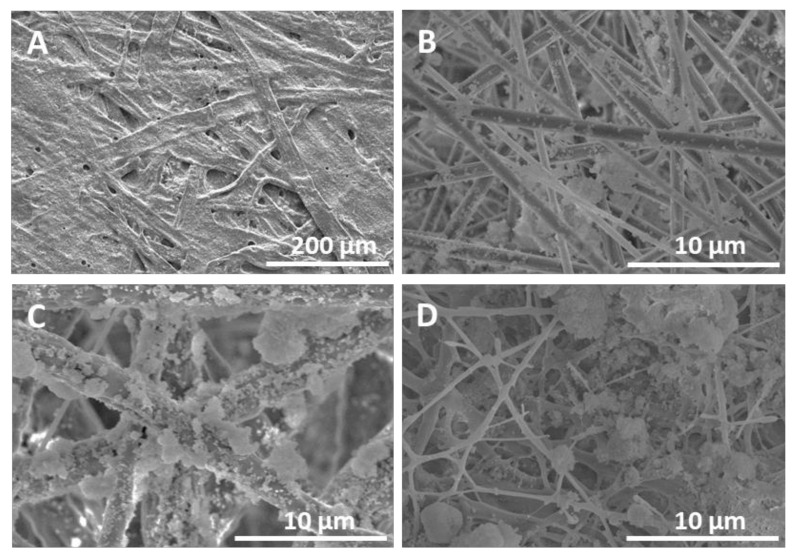
Field emission scanning electron microscope (FE-SEM) images of the multilayer structures based on paper, electrospun polylactide (PLA), poly(ε-caprolactone) (PCL), and poly(3-hydroxybutyrate-co-3-hydroxy-valerate) (PHBV) mats, and electrosprayed hydrophobic silica microparticles. (**A**): as-received paper; (**B**): Paper/PLA/SiO_2_; (**C**): Paper/PCL/SiO_2_; (**D**): Paper/PHBV/SiO_2_.

Figure 5 presents the morphology of the non-coated and coated paper samples analyzed under FE-SEM. For the non-coated paper (Figure 5A), it can be observed that the material is composed of thick cellulose fibers with irregular morphology, as is well-known. As for the case of electrospun/electrosprayed coated samples, it is important to notice that, while both PLA (see Figure 5B) and PCL (see Figure 5C) yielded more uniform fibers, the PHBV coating (see Figure 5D) presented somewhat more irregular morphology. This could be attributed to the lower polymer concentration optimally required for this polymer to achieve stable electrospinning. Regarding the hydrophobic silica microparticles deposition by electrospraying onto the PLA and PCL layers, it is possible to observe coated fibers with microparticles and also some particle agglomerates (see Figure 5B,C). However, PLA and PCL fibers seemed to accommodate the particles in a more homogeneous manner than its PHBV counterpart (see Figure 5D), where more silica particle agglomerates were apparent.

Mean fiber diameters of the PLA, PCL and PHBV electrospun coatings were measured from the FE-SEM images and gathered in Table 4. PLA and PHBV polymers produced fibers with mean diameters below one micron, whereas for the PCL, fibers around 3 µm were obtained. Lasprilla et al. also obtained PCL fibers with a mean diameter of 3.0 µm using the electrospinning technology [12]. Melendez-Rodriguez et al. and Pardo-Figuerez et al. reported mean fiber diameters of 1.5 µm for PLA and 1.32 µm for PHBV processed via electrospinning [11,44]. These differences may be due to the use of different conditions, that is, polymer grade and concentration, solvent, flow rate, voltage, tip-to-collector distance, temperature, and relative humidity [45,46]. Indeed, fiber diameter is known to increase with increasing polymer concentration and flow rate, while fiber diameter can decrease with longer tip-to-collector distance and increasing voltage [47].

### 3.3. Thermal Treatment Optimization

In order to optimize the annealing treatment to promote interlayer adhesion, a thermal analysis of the electrospun PLA, PCL, and PHBV fiber mats were carried out using DSC. The results indicated that the electrospun PLA samples showed a Tm value at 170 °C, in agreement with previous work [48]. In the case of the PCL, the Tm value was at 59 °C also in agreement with the previous literature [12], and the Tm of PHBV was 153 °C, in a single peak, again in agreement with a previous study [49]. 

**Figure 6:**
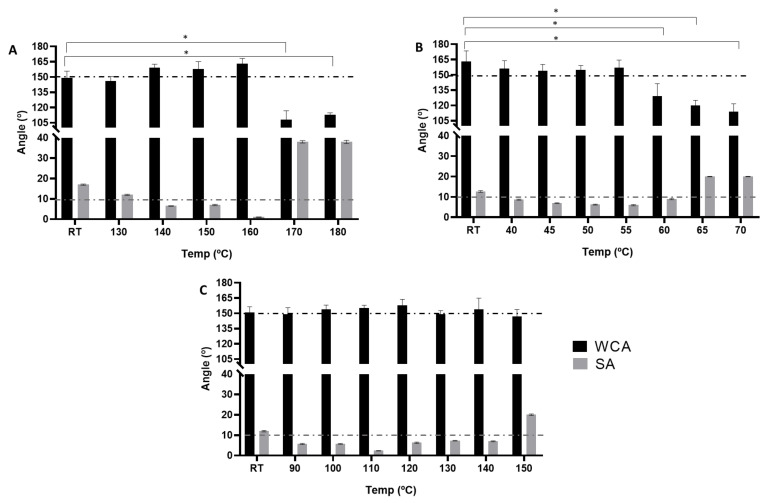
Apparent water contact angle (WCA) and sliding angle (SA) before and after thermal post-treatment at different temperatures of the multilayer structures based on paper, electrospun polylactide (PLA), poly(ε-caprolactone) (PCL), and poly(3-hydroxybutyrate-co-3-hydroxy-valerate) (PHBV) mats, and electrosprayed hydrophobic silica microparticles. (**A**) Paper/PLA/SiO_2_ for 20 s; (**B**) Paper/PCL/SiO_2_ for 20 s; (**C**) Paper/PHBV/SiO_2_ for 20 s. Black line at 150 °C represents the minimum WCA and grey line at 10 °C the max SA to be defined as superhydrophobic. RT means at room temperature. Asterisks indicate statistical differences between the room temperature and annealed samples (* *p* < 0.05).

Different thermal post-treatments were carried out for each multilayer structure in order to determine the most optimal conditions to achieve a strong adhesion between layers as well as the maximum super-hydrophobicity, and to enhance its barrier properties. In particular, different temperatures (Table 2) were evaluated for each material and compared with a non-thermally treated comparable sample. The temperature ranges were selected according to the Tm values of PLA, PCL, PHBV, and previous works carried out in our group [11,12,44]. Previous studies suggested that electrospun fibers alone did not yield superhydrophobic behavior, reaching values of WCA of 96° and 128° for PLA and PCL, respectively [11,12]. In this study, similar results of WCA were obtained for electrospun fibers of PLA and PCL fibers, i.e., 108° and 126°, whereas for PHBV, a value of 122° was measured. As stated above, superhydrophobic characteristics are defined when the apparent water contact angle (WCA or θ *) >150° and the sliding angle (SA) < 10°. Values in these ranges were only achieved by additional deposition of silica (see Figure 6).

To obtain superhydrophobic properties, the required thickness of the electrosprayed silica microparticles layers was, according to Table 3, of 2.2, 5.0, and 1.6 µm for the coatings on PLA, PCL, and PHBV, respectively. As it can be observed, the SiO_2_ amount needed in the PCL layer to obtain superhydrophobic properties was higher than for the other polymers. When we applied a thinner coating of PCL fibers but maintained the optimized quantity of silica, the superhydrophobic behavior was not retained (results not shown). This indicates that there needs to be an optimal quantity of fibers and particles for each material for this property to be displayed. The reason for PCL to require a thicker layer of fibers and particle may be related to the higher fiber size attained.

Figure 6A shows that the coated paper with PLA/SiO_2_ presented, prior to annealing, a WCA of 149°. As the annealing temperature increased up to 160 °C, the WCA values increased to 163°, and the values of SAs were reduced from 12° to < 2° (*p* < 0.05). It can also be observed that, from 160 to 180 °C, the performance was totally different, having WCA values of < 115° and SA > 38°, suggesting that the coating repelling properties, such as surface roughness, diminished due to melting of the fibers. This effect can be followed by evolution of the surface morphology as a function of the annealing temperature, as determined by FE-SEM, of the various multilayer structures (see Supplementary Material Figure S1).

A similar tendency can be observed for the PCL/SiO_2_ coatings in Figure 6B, where the WCA values remained higher than 150°, while the SA values were reduced from 12° to < 6° until reaching 55 °C of thermal treatment. Annealing temperatures above that led to a drop in superhydrophobicity (*p* < 0.05). This effect can be ascribed to the fact that the PCL fibers melted above 55 °C, as supported by FE-SEM top views of the multilayer structures (see Supplementary Material Figure S2F–H), in which a reduction in surface roughness was seen to take place.

Regarding PHBV/SiO_2_, the hydrophobic behavior was nearly the same as PLA and PCL, but the differences in the WCA values among the different annealed samples were smaller, with values ranging from 147 to 157°. The highest WCA value was obtained when annealing was performed at 120 °C, which is a temperature well below the T_m_ of this microbial copolyester. The resultant SAs values were also, in almost all cases, <10° with two exceptions, that is, prior to thermal treatment (SA 12°) and at 150 °C (SA 20°). The former value is ascribed to the higher porosity of the surface, whereas the latter can be attributed to proximity to the melting point, which led to reduced porosity. This phenomenon was also supported by FE-SEM micrographs (see Supplementary Material Figure S3), in which the presence of the fibers can be seen at low temperatures while the interfiber morphology collapsed at the highest temperature.

**Figure 7:**
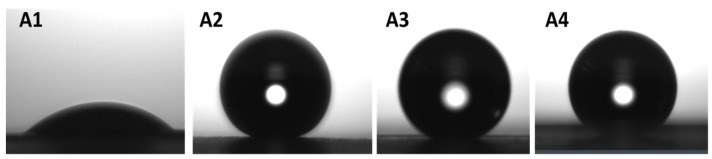
Shape of the water droplets after 30 s on: (**A1**): Paper, (**A2**): Paper/PLA/SiO2 annealed at 160 °C for 20 s, (**A3**): Paper/PCL/SiO_2_ annealed at 55 °C for 20 s, (**A4**): Paper/PHBV/SiO_2_ annealed at 120 °C for 20 s.

Previous works carried out in our group have already reported similar results regarding the optimal annealing temperatures for electrospun fibers of PLA and PCL on different substrates, and electrospun monolayers of a similar PHBV [11,12,44]. Based on the values obtained from the thermal treatment optimization, it can be concluded that the most promising samples with the best superhydrophobic features were Paper/PLA/SiO_2_ at 160 °C, Paper/PCL/SiO_2_ at 55 °C, and Paper/PHBV/SiO_2_ at 120 °C. Figure 7 shows the shape of the water droplets deposited over the non-coated paper, and the different applied coatings composed of biopolymers and silica microparticles, further highlighting the improvement on the superhydrophobic characteristics of the generated biocoatings. In these experiments, the paper substrate was seen to drop from a CA of 70° to nearly 0° after 60 s of contact time, confirming the expected very low water barrier of the paper substrate, whereas the coated papers retained the CA of the water droplets over time.

### 3.4. Barrier Properties

The barrier properties to water vapor represent some of the most important parameters in food packaging applications [37]. Table 5 gathers the values of the water vapor permeance of the selected samples of each biopolymer together with the ones treated at the lowest and highest annealing temperatures for Paper/PLA/SiO_2_, Paper/PCL/SiO_2_, and Paper/PHBV/SiO_2_. The goal was to analyze the water barrier properties of these coatings compared with the non-coated paper and evaluate if the selected ones, which are based on superior superhydrophobic properties, also presented improved vapor barrier performance.

The water vapor permeance obtained for the uncoated paper was of 2.35 × 10^−9^ kg·Pa^−1^·s^−1^·m^−2^, a value that is in the same order of magnitude as described in a previous work, in which a virgin experimental uncoated cellulose paper substrate was used [50]. The Paper/PLA/SiO_2_ multilayer annealed at 130, 160, and 180 °C yielded a water vapor permeance of 1.88, 1.67, and 1.98 × 10^−9^ kg·Pa^−1^·s^−1^·m^−2^, respectively. Similar results were obtained for Paper/PCL/SiO_2_, where the permeance was 1.82, 1.55, and 1.67 × 10^−9^ kg·Pa^−1^·s^−1^·m^−2^, for samples treated at 40, 55, and 70 °C, respectively, and for Paper/PHBV/SiO_2_ with values of 1.81, 1.52, and 1.62 × 10^−9^ kg·Pa^−1^·s^−1^·m^−2^, for samples annealed at 90, 120, and 150 °C, respectively. From the results, it can be concluded that the optimal annealing conditions in terms of superhydrophobicity also yielded the lowest water permeance. This must be the result of both reduced water solubility and enhanced morphology cohesion, leading to a reduced diffusion. Interestingly, even though annealing at the highest temperature yielded a much more reduced porosity, since water contact angle decreased, the water vapor barrier effect also decreased, suggesting that a reduction in solubility is key for the water vapor barrier performance of these materials. Therefore, the results obtained here clearly suggest that the selected samples, in terms of superhydrophobic features, also presented the lowest water permeance values. It is also worthy to note that the PHBV-based coating was slightly more efficient in reducing water vapor permeance, even though it presented the lowest thickness, most likely due to the higher barrier performance of PHBV in comparison to PLA and other biodegradable aliphatic polyesters, as previously reported [51].

### 3.5. Tape Peel-Off Adhesion Test

The tape peel-off adhesion test is typically used to assess the qualitative adhesion strength of coatings to substrates. After the test, WCA and SA were measured again. As recommended in the literature, the adhesive tape was forced to strongly stick on the coated surface by pressing with the hand, before the tape was peeled off from the substrate surface [16,32,52]. From the results, while some stains were left on the black tape for the case of Paper/PLA/SiO_2_ and Paper/PCL/SiO_2_ multilayers, this was seen to occur to a much lesser extent the case for the Paper/PHBV/SiO_2_ multilayer.

**Figure 8:**
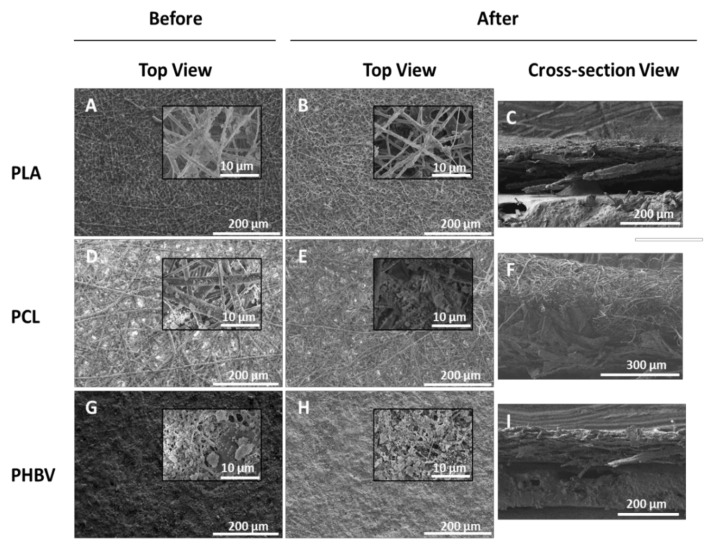
Top and cross-section views images by field emission scanning electron microscope (FE-SEM) before and after performing the tape peel-off adhesion test of the multilayer structures based on paper, electrospun polylactide (PLA), poly(ε-caprolactone) (PCL), and poly(3-hydroxybutyrate-co-3-hydroxy-valerate) (PHBV) mats, and electrosprayed hydrophobic silica microparticles: (**A**–**C**) Paper/PLA/SiO_2_ annealed at 160 °C; (**D**–**F**) Paper/PCL/SiO_2_ annealed at 55 °C; and (**G**–**I**) Paper/PHBV/SiO_2_ annealed at 120 °C.

In order to ascertain the morphological effect of the tape adhesion test on the multilayers, the morphology of the samples before and after the peeling off test were analyzed by FE-SEM. Figure 8 illustrates the top view images of the coatings, where there were no apparent visible differences in terms of roughness and morphology before and after the test. However, the multilayer cross-section view revealed, after the peeling process, a somewhat less compact coating integrity for the samples prepared with PLA (see Figure 8C) and PCL (see Figure 8F). Regarding the cross-sectional view of the multilayer based on PHBV, the coating was seen to be significantly less affected after the tape adhesion test (see Figure 8I). This suggests that the latter biopolymer system resulted in stronger interlayer adhesion after the annealing process, which could, in turn, be caused by the particular thermal and mechanical properties of the polymer and, perhaps, also by the particular lowest fiber cross-section and morphological arrangements generated (see the Supplementary Material).

Regarding the WCA and SA values, after the peeling-off test, the three coated samples presented a dissimilar behavior (Table 6). Thus, the paper coated with PLA/SiO_2_ showed similar WCA values (163–166°) but SA drastically increased from <2° to >45° after the tape adhesion test. This phenomenon is termed in the literature as the “petal effect”, where the static performance, that is, WCA, remains high but, at the same time, a water droplet cannot roll off the surface. This effect implies that these surfaces were still superhydrophobic but showed a higher tortuous topology for the water droplets to slide [16,18]. In the case of the Paper/PCL/SiO_2_ multilayer, the WCA value was reduced from 169° to 147° and also the value of SA increased from 3° to 24°. However, for the Paper/PHBV/SiO_2_ multilayer, the values remained similar WCA (~158°) and SA (2–3°) before and after the tape peel-off test, confirming its higher interlayer adhesion and mechanical resistance. The differences among the three coated samples depended on their intrinsic roughness and their capacity to maintain the hierarchical structure of the biopolymer-silica surface. The coated paper using PHBV presents really promising results since it is able to maintain the same level of superhydrophobicity even after the peel-off test, suggesting stronger adhesion properties between layers for this particular biopaper. 

### 3.6. Viscous Food Super-Repelling Properties 

The viscous food super-repelling properties of the paper-based multilayers were evaluated using yogurt, custard, ketchup, and mayonnaise. Table 7 shows the contact angle (CA) and sliding angle (SA) of the uncoated paper and the multilayers Paper/PLA/SiO_2_ annealed at 160 °C, Paper/PCL/SiO_2_ annealed at 55 °C, and Paper/PHBV/SiO_2_ annealed at 120 °C. In the case of yogurt, the uncoated paper presented a CA of ~96° and SA > 45°. In the coated structures, the CA highly increased, reaching values of ~150° or higher, and the SA values were reduced to approximately 10°, which implied that the coatings developed present high super-repellency to yogurt, which easily rolled-off.

The performance of the coatings to custard showed some differences. Although the CA values obtained with the three coatings yielded values of ~150° or even higher (whereas the uncoated paper had a value of 56°), the SA values widely varied as a function of the coating type. Sliding angles higher to 45° were obtained for the Paper/PLA/SiO_2_ and Paper/PCL/SiO_2_ multilayers but the Paper/PHBV/SiO_2_ multilayer interestingly presented a value of 24°. As previously described, the Paper/PLA/SiO_2_ and Paper/PCL/SiO_2_ multilayers exhibited the “petal effect”, with high static contact angles but also high adhesion between custard droplets and the surface. However, the Paper/PHBV/SiO_2_ multilayer is considered to keep the Cassie–Baxter state, leading to low sliding angle values and, hence, custard droplets easily rolled-off through the surface. In this regard, it is worthy to mention that, compared to yogurt, custard composition is denser and fattier, and, normally, emulsifiers and stabilizers are employed in its preparation, which can explain these differences. Indeed, it has been reported that additives, such as emulsifiers and stabilizers, can affect the Cassie state of rough surfaces [53].

Regarding ketchup and mayonnaise, the contact angle could not be appropriately measured due to the high viscosity of these foodstuffs, obtaining no significant differences between the uncoated and the coated papers when these food materials were evaluated. Similarly to what was observed with custard, this result can be ascribed to the fact that the ingredients present in their composition can influence the performance of the coated papers developed in this work.

### 3.7. Effect of Food Product Temperature on Super-Repellent Performance

The effect of temperature on the roll-off capacity of the multilayers was also evaluated by employing the same food products at 22 °C and 50 °C since the packaging of food products at an industrial scale was carried out at temperatures higher than 40–45 °C. For that purpose, the roll-off capacity was measured in samples that were fixed on a flat surface at 45° angle of tilt respect to the horizontal. The roll-off angle values can be influenced by the dispensing height and the size of the droplets [16], so the height was fixed at 3 cm from the surface but the droplet volume depended on the particular foodstuff viscosity (see Experimental part). As shown in Table 8, the non-coated paper showed no roll-off capacity under the experimental conditions used for any of the food products and temperatures, while the coated papers presented different performances. The three coated papers were able to roll-off water and yogurt at both temperatures, whereas none of them was able to successfully perform the roll-off capacity with ketchup and mayonnaise. Interestingly, regarding custard, the performance was really different depending on the coated sample. The Paper/PLA/SiO_2_ multilayer presented a slight capacity to roll-off custard at both temperatures. In regard to the Paper/PCL/SiO_2_ multilayer, the effect was good at 22 °C but not at 50 °C. However, this sample was able to roll-off custard at 50 °C when the angle was increased to 80° (result not reported in Table 8). Regarding the Paper/PHBV/SiO_2_ multilayer, its performance was very similar, regardless of the food temperature used, since the roll-off capacity was very good at both temperatures. These results point out that the coated paper using PHBV can maintain its super-repellent properties for food applications even at temperatures higher than 50 °C. The reason for this could be ascribed to the known enhanced thermal resistance of PHBV, compared to PCL (low melting point) and PLA (with T_g_ around 55 °C) [44].

## 4. Conclusions

A super-repelling coated paper suitable for food contact applications was developed by EDHP using electrospun PLA, PCL, and PHBV, and electrosprayed food contact hydrophobic silica. The ultrathin electrospun biopolymer fibers were first deposited onto uncoated cellulose paper by electrospinning and, thereafter, hydrophobic silica was electrosprayed on top of the electrospun mat. The resultant coated papers were then post-treated at different annealing temperatures for 20 s to promote binding by interfibers coalescence between the layers. The optimal conditions were attained at 160 °C, 55 °C, and 120 °C for PLA, PCL, and PHBV, respectively. The results showed that the WCA values were higher than 155° while the values of SA were lower than 10° in all cases. The water vapor permeance compared to the uncoated cellulose paper (2.35 × 10^−9^ kg·Pa^−1^·s^−1^·m^−2^) was reduced in all cases, showing that the Paper/PHBV/SiO_2_ multilayer obtained the best barrier properties among them (1.52 × 10^−9^ kg·Pa^−1^·s^−1^·m^−2^). In terms of the adhesion strength of the coating by the so-called peeling-off test, the Paper/PLA/SiO_2_ and Paper/PCL/SiO_2_ multilayers showed somewhat weaker adhesion than the Paper/PHBV/SiO_2_ multilayer, which maintained after the test the highest WCA (>155°) and lowest SA (<5°) values. Super-repellency was also evaluated with several food products, that is, yogurt, custard, ketchup, and mayonnaise, at both 22 °C or heated at 50 °C, measuring their CA and SA values as well as their roll-off capacity when the surface was tilted at 45°. All the here-developed coatings obtained high CA and relatively low SA values, and they easily rolled off with yogurt at both temperatures. In the case of custard, the performance varied as a function of the coatings, with the Paper/PHBV/SiO_2_ multilayer being the most promising since it kept a high CA (154°) and low SA (24°) values.

The generated super-repellent multilayers based on paper coated with electrospun PLA, PCL, or PHBV and electrosprayed hydrophobic silica microparticles herein reported show the potential use in food packaging applications to reduce food waste, improve environmental friendliness in comparison with polyolefin coatings by using proven compostable biopolymers as coating materials, and also enhance the water barrier. Among all the prepared multilayers, the super-repellent paper developed using biowaste-derived PHBV showed the best performance in terms of hydrophobicity, barrier properties, and self-cleaning properties for fatty and dense food products, such as yogurt and custard. The greater performance of the Paper/PHBV/SiO_2_ multilayer compared to the others polymers may be due to better interlayer adhesion which, in turn, could be due to the particular physico-chemical properties of the polymer, i.e., higher thermal resistance, and fiber morphology generated. 

## Figures and Tables

**Table 1:** Electrospinning parameters for polylactide (PLA), poly(ε-caprolactone) (PCL), and poly(3-hy-droxybutyrate-co-3-hydroxy-valerate) (PHBV) and the electrospraying of the hydrophobic silica particles (SiO_2_). The substrate was, in all cases, cellulose paper. Dz: Distance needle to collector.

Sample	Polymer or Particle Content (wt %)	Solvent (vol./vol.)	Flow Rate (mL/h)	Needle Gauge (G)	Dz (cm)	V+ Injector/V− Collector (kV)	Relative Humidity (%)	Temperature (°C)
PLA	12.5	DCM/TFE 50:50	10	22	21	19.1/−5.4	27	25
PCL	10	TCM/MeOH 8:2	20	22	21	19.0/−5.5	43	25
PHBV	2	TCM/BuOH 75:25	30	23	21	24.5/−5.5	41	25
PLA/SiO_2_	0.75	BuOH	10	18	21	19.7/−5.5	29	25
PCL/SiO_2_	0.75	BuOH	10	18	21	19.7/−5.5	35	25
PHBV/SiO_2_	0.75	BuOH	10	18	21	19.7/−5.5	36	25

**Table 2:** Conditions employed for the thermal post-treatment of Paper/PLA/SiO_2_, Paper/PCL/SiO_2_, and Paper/PHBV/SiO_2_ samples.

Sample	Temperature (°C)							Time (s)
Paper/PLA/SiO_2_	130	140	150	160	170	180	-	20
Paper/PCL/SiO_2_	40	45	50	55	60	65	70	20
Paper/PHBV/SiO_2_	90	100	110	120	130	140	150	20

**Table 3:** Thickness of the multilayer structures based on paper, electrospun polylactide (PLA), poly(ε-caprolactone) (PCL), and poly(3-hydroxybutyrate-co-3-hydroxy-valerate) (PHBV) mats, and electrosprayed hydrophobic silica microparticles.

Sample	Thickness (µm)
Paper	77.9 ± 2.0
Paper/PLA	98.5 ± 1.0
Paper/PCL	112.5 ± 3.0
Paper/PHBV	92.2 ± 2.1
Paper/PLA/SiO_2_	100.7 ± 3.2
Paper/PCL/SiO_2_	117.5 ± 2.5
Paper/PHBV/SiO_2_	93.8 ± 0.8

**Table 4:** Fiber diameter of PLA, PCL and PHBV electrospun coatings on paper.

Sample	Fiber Diameter (µm)
Paper/PLA	0.80 ± 0.21
Paper/PCL	3.22 ± 1.32
Paper/PHBV	0.39 ± 0.19

**Table 5:** Mean thicknesses and water vapor permeance of non-coated and the multilayer structures based on paper, electrospun polylactide (PLA), poly(ε-caprolactone) (PCL), and poly(3-hydroxybutyrate-co-3-hydroxy-valerate) (PHBV) mats, and electrosprayed hydrophobic silica microparticles annealed at different temperatures.

Sample	Thickness (µm)	Water Vapor Permeance × 10^−9^ (kg·Pa^−1^·s^−1^·m^−2^)
Paper	77.9 ± 2.0	2.35 ± 0.04
Paper/PLA/SiO_2_ at 130 °C	103.4 ± 1.9	1.88 ± 0.30 ^a^
Paper/PLA/SiO_2_ at 160 °C	103.6 ± 2.0	1.67 ± 0.15 ^a^
Paper/PLA/SiO_2_ at 180 °C	99.0 ± 2.0	1.98 ± 0.06 ^a^
Paper/PCL/SiO_2_ at 40 °C	118.5 ± 2.8	1.82 ± 0.07 ^a^
Paper/PCL/SiO_2_ at 55 °C	115.4 ± 3.3	1.55 ± 0.09 ^b^
Paper/PCL/SiO_2_ at 70 °C	94.9 ± 2.7	1.67 ± 0.13 ^a,b^
Paper/PHBV/SiO_2_ at 90 °C	94.4 ± 2.6	1.81 ± 0.12 ^a^
Paper/PHBV/SiO_2_ at 120 °C	90.8 ± 2.4	1.52 ± 0.16 ^b^
Paper/PHBV/SiO_2_ at 150 °C	90.5 ± 2.4	1.62 ± 0.06 ^a,b^

^a,b^ Different letters in the same column for the same polymer (PLA, PCL, and PHBV) mean significant difference among the samples (*p* < 0.05).

**Table 6:** Apparent water contact angle (WCA) and sliding angle (SA) of the multilayer structures based on paper, electrospun polylactide (PLA), poly(ε-caprolactone) (PCL), and poly(3-hydroxybutyrate-co-3-hydroxy-valerate) (PHBV) mats, and electrosprayed hydrophobic silica microparticles before and after the tape adhesion test of Paper/PLA/SiO_2_ annealed at 160 °C, Paper/PCL/SiO_2_ annealed at 55 °C, and Paper/PHBV/SiO_2_ annealed at 120 °C.

	Before		After	
Sample	WCA (°)	SA (°)	WCA (°)	SA (°)
Paper/PLA/SiO_2_ at 160 °C	163 ± 3 ^a^	<2	166 ± 7 ^a^	>45
Paper/PCL/SiO_2_ at 55 °C	169 ± 3 ^a^	3	147 ± 2 ^b^	24
Paper/PHBV/SiO_2_ at 120 °C	157 ± 3 ^a^	2	160 ± 3 ^a^	3

^a,b^ Different letters in the same line for the WCA mean significant difference among the samples (*p* < 0.05).

**Table 7:** Contact angle (CA) and sliding angle (SA) for yogurt, custard, ketchup, and mayonnaise of the uncoated paper and the multilayer structures based on paper, electrospun polylactide (PLA), poly(ε-caprolactone) (PCL), and poly(3-hydroxybutyrate-co-3-hydroxy-valerate) (PHBV) mats, and electrosprayed hydrophobic silica microparticles of Paper/PLA/SiO_2_ annealed at 160 °C, Paper/PCL/SiO_2_ annealed at 55 °C, and Paper/PHBV/SiO_2_ annealed at 120 °C. For ketchup and mayonnaise, the contact angle could not be measured.

	Paper		Paper/PLA/SiO_2_		Paper/PCL/SiO_2_		Paper/PHBV/SiO_2_	
Sample	CA (°)	SA (°)	CA (°)	SA (°)	CA (°)	SA (°)	CA (°)	SA (°)
Yogurt	96 ± 3 ^a^	>45	148 ± 10 ^b^	10.4	159 ± 7 ^b^	8	166 ± 6 ^b^	14
Custard	56 ± 11 ^a^	>45	143 ± 7 ^b^	>45	155 ± 6 ^b^	>45	154 ± 4 ^b^	24
Ketchup	-	>45	-	>45	-	>45	-	>45
Mayonnaise	-	>45	-	>45	-	>45	-	>45

^a,b^ Different letters in the same line for the CA mean significant difference among the Paper- Paper/PLA/SiO_2_, Paper- Paper/PCL/SiO_2_ and Paper- Paper/PHBV/SiO_2_ samples (*p* < 0.05).

**Table 8:** Roll-off capacity at 45° angle of the uncoated paper and the multilayer structures based on paper, electrospun polylactide (PLA), poly(ε-caprolactone) (PCL), and poly(3-hydroxybutyrate-co-3-hydroxy-valerate) (PHBV) mats, and electrosprayed hydrophobic silica microparticles Paper/PLA/SiO_2_ annealed at 160 °C, Paper/PCL/SiO_2_ annealed at 55 °C, and Paper/PHBV/SiO_2_ annealed at 120 °C using water, yogurt, custard, ketchup, and mayonnaise at 22 and 50 °C.

		Rolling off Capacity from the Sample			
Sample	Temperature (°C)	Paper	Paper/PLA/SiO_2_	Paper/PCL/SiO_2_	Paper/PHBV/SiO_2_
Water	22	NO	YES	YES	YES
	50	NO	YES	YES	YES
Yogurt	22	NO	YES	YES	YES
	50	NO	YES	YES	YES
Custard	22	NO	SLIGHTLY	YES	YES
	50	NO	SLIGHTLY	NO	YES
Ketchup	22	NO	NO	NO	NO
	50	NO	NO	NO	NO
Mayonnaise	22	NO	NO	NO	NO
	50	NO	NO	NO	NO

## Data Availability

The data presented in this work are available upon request from the corresponding author.

## Supplementary Materials

The following are available online at https://www.mdpi.com/article/10.3390/nano11123354/s1, Figure S1: Field emission scanning electron microscope (FE-SEM) images of the multilayer structures based on paper, electrospun polylactide (PLA) and electrosprayed hydrophobic silica microparticles (Paper/PLA/SiO_2_) at different annealing temperatures for 20 s. A: No annealing; B: 130 °C; C: 140 °C; D: 150 °C; E: 160 °C; F: 170 °C; G: 180 °C. Figure S2: Field emission scanning electron microscope (FE-SEM) images of the multilayer structures based on paper, electrospun poly(ε-caprolactone) (PCL) and electrosprayed hydrophobic silica microparticles (Paper/PCL/SiO_2_) at different annealing temperatures for 20 s. A: No annealing; B: 40 °C; C: 45 °C; D: 50 °C; E: 55 °C; F: 60 °C; G: 65 °C; H: 70 °C. Figure S3: Field emission scanning electron microscope (FE-SEM) images of the multilayer structures based on paper, electrospun poly(3-hydroxybutyrate-co-3-hydroxy-valerate) (PHBV), and electrosprayed hydrophobic silica microparticles (Paper/PHBV/SiO_2_) at different annealing temperatures for 20 s: A: No annealing; B: 90 °C; C: 100 °C; D: 110 °C; E: 120 °C; F: 130 °C; G: 140 °C; H: 150 °C.

## References

[1] Li H., He Y., Yang J., Wang X., Lan T., Peng L. (2019). Fabrication of Food-Safe Superhydrophobic Cellulose Paper with Improved Moisture and Air Barrier Properties. Carbohydr. Polym..

[2] Deshwal G.K., Panjagari N.R., Alam T. (2019). An Overview of Paper and Paper Based Food Packaging Materials: Health Safety and Environmental Concerns. J. Food Sci. Technol..

[3] Ogihara H., Xie J., Okagaki J., Saji T. (2012). Simple Method for Preparing Superhydrophobic Paper: Spray-Deposited Hydrophobic Silica Nanoparticle Coatings Exhibit High Water-Repellency and Transparency. Langmuir.

[4] Martins V.D.F., Cerqueira M.A., Fuciños P., Garrido-Maestu A., Curto J.M.R., Pastrana L.M. (2018). Active Bi-Layer Cellulose-Based Films: Development and Characterization. Cellulose.

[5] Balu B., Breedveld V., Hess D.W. (2008). Fabrication of “Roll-off” and “Sticky” Superhydrophobic Cellulose Surfaces via Plasma Processing. Langmuir.

[6] Dias V.M., Kuznetsova A., Tedim J., Yaremchenko A.A., Zheludkevich M.L., Portugal I., Evtuguin D.V. (2015). Silica-Based Nanocoating Doped by Layered Double Hydroxides to Enhance the Paperboard Barrier Properties. World J. Nano Sci. Eng..

[7] Zhao X., Cornish K., Vodovotz Y. (2020). Narrowing the Gap for Bioplastic Use in Food Packaging: An Update. Environ. Sci. Technol..

[8] Cunha A.G., Gandini A. (2010). Turning Polysaccharides into Hydrophobic Materials: A Critical Review. Part 1. Cellulose. Cellulose.

[9] European Bioplastics (2020). https://www.european-bioplastics.org/europes-new-circular-economy-action-plan-needs-to-link-bioeconomy-and-circular-economy.

[10] Arrieta M.P., Peponi L., López D., López J., Kenny J.M., Grumezescu A.M. (2017). 12—An overview of nanoparticles role in the improvement of barrier properties of bioplastics for food packaging applications. Food Packaging.

[11] Pardo-Figuerez M., López-Córdoba A., Torres-Giner S., Lagaron J. (2018). Superhydrophobic Bio-Coating Made by Co-Continuous Electrospinning and Electrospraying on Polyethylene Terephthalate Films Proposed as Easy Emptying Transparent Food Packaging. Coatings.

[12] Lasprilla-Botero J., Torres-Giner S., Pardo-Figuerez M., Álvarez-Láinez M., Lagaron J.M. (2018). Superhydrophobic Bilayer Coating Based on Annealed Electrospun Ultrathin Poly(ε-Caprolactone) Fibers and Electrosprayed Nanostructured Silica Microparticles for Easy Emptying Packaging Applications. Coatings.

[13] Wen G., Huang J.X., Guo Z.G. (2019). Energy-Effective Superhydrophobic Nanocoating Based on Recycled Eggshell. Colloids Surf. A Physicochem. Eng. Asp..

[14] Zhang C., Kalulu M., Sun S., Jiang P., Zhou X., Wei Y., Jiang Y. (2019). Environmentally Safe, Durable and Transparent Superhydrophobic Coating Prepared by One-Step Spraying. Colloids Surf. A Physicochem. Eng. Asp..

[15] Zhao X., Hu T., Zhang J. (2018). Superhydrophobic Coatings with High Repellency to Daily Consumed Liquid Foods Based on Food Grade Waxes. J. Colloid Interface Sci..

[16] Milionis A., Loth E., Bayer I.S. (2016). Recent Advances in the Mechanical Durability of Superhydrophobic Materials. Adv. Colloid Interface Sci..

[17] Liu B.-Y., Xue C.-H., An Q.-F., Jia S.-T., Xu M.-M. (2019). Fabrication of Superhydrophobic Coatings with Edible Materials for Super-Repelling Non-Newtonian Liquid Foods. Chem. Eng. J..

[18] Karapanagiotis I., Grosu D., Aslanidou D., Aifantis K.E. (2015). Facile Method to Prepare Superhydrophobic and Water Repellent Cellulosic Paper. J. Nanomater..

[19] Razavi S.M.R., Oh J., Haasch R.T., Kim K., Masoomi M., Bagheri R., Slauch J.M., Miljkovic N. (2019). Environment-Friendly Antibiofouling Superhydrophobic Coatings. ACS Sustain. Chem. Eng..

[20] Nyström D., Lindqvist J., Östmark E., Hult A., Malmström E. (2006). Superhydrophobic Bio-Fibre Surfaces via Tailored Grafting Architecture. Chem. Commun..

[21] Li S., Zhang S., Wang X. (2008). Fabrication of Superhydrophobic Cellulose-Based Materials through a Solution-Immersion Process. Langmuir.

[22] Quan C., Werner O., Wågberg L., Turner C. (2009). Generation of Superhydrophobic Paper Surfaces by a Rapidly Expanding Supercritical Carbon Dioxide–Alkyl Ketene Dimer Solution. J. Supercrit. Fluids.

[23] Zhang Y., Xu Q., Jiang B., Ma Z. (2020). Flame-Retardant Paper with Robust Hydrophobicity Enabled by Perfluorodecane Doped SiO_2_ Nanofibers. J. Sol-Gel Sci. Technol..

[24] Torres-Giner S., Pérez-Masiá R., Lagaron J.M. (2016). A Review on Electrospun Polymer Nanostructures as Advanced Bioactive Platforms. Polym. Eng. Sci..

[25] Wang C.-X., Zhang X.-F. (2020). A Non-Particle and Fluorine-Free Superhydrophobic Surface Based on One-Step Electrodeposition of Dodecyltrimethoxysilane on Mild Steel for Corrosion Protection. Corros. Sci..

[26] Rivero P.J., Iribarren A., Larumbe S., Palacio J.F., Rodríguez R. (2019). A Comparative Study of Multifunctional Coatings Based on Electrospun Fibers with Incorporated ZnO Nanoparticles. Coatings.

[27] Fabra M.J., López-Rubio A., Cabedo L., Lagaron J.M. (2016). Tailoring Barrier Properties of Thermoplastic Corn Starch-Based Films (TPCS) by Means of a Multilayer Design. J. Colloid Interface Sci..

[28] Irzh A., Ghindes L., Gedanken A. (2011). Rapid Deposition of Transparent Super-Hydrophobic Layers on Various Surfaces Using Microwave Plasma. ACS Appl. Mater. Interfaces.

[29] Gong X., He S. (2020). Highly Durable Superhydrophobic Polydimethylsiloxane/Silica Nanocomposite Surfaces with Good Self-Cleaning Ability. ACS Omega.

[30] Fu J., Yang F., Guo Z. (2019). Facile Fabrication of Superhydrophobic Filter Paper with High Water Adhesion. Mater. Lett..

[31] Wang Q., Xie D., Chen J., Liu G., Yu M. (2020). Superhydrophobic Paper Fabricated via Nanostructured Titanium Dioxide-Functionalized Wood Cellulose Fibers. J. Mater. Sci..

[32] Latthe S.S., Sutar R.S., Kodag V.S., Bhosale A.K., Kumar A.M., Kumar Sadasivuni K., Xing R., Liu S. (2019). Self-Cleaning Superhydrophobic Coatings: Potential Industrial Applications. Prog. Org. Coat..

[33] Sriramulu D., Reed E.L., Annamalai M., Venkatesan T.V., Valiyaveettil S. (2016). Synthesis and Characterization of Superhydrophobic, Self-Cleaning NIR-Reflective Silica Nanoparticles. Sci. Rep..

[34] Zhang P., Lv F.Y. (2015). A Review of the Recent Advances in Superhydrophobic Surfaces and the Emerging Energy-Related Applications. Energy.

[35] Commission Regulation (EU) https://eur-lex.europa.eu/legal-content/EN/ALL/?uri=celex%3A32011R0010.

[36] Volova T.G., Kalacheva G.S. (2005). The Synthesis of Hydroxybutyrate and Hydroxyvalerate Copolymers by the Bacterium Ralstonia Eutropha. Mikrobiologiya.

[37] Melendez-Rodriguez B., Torres-Giner S., Aldureid A., Cabedo L., Lagaron J.M. (2019). Reactive Melt Mixing of Poly(3-Hydroxybutyrate)/Rice Husk Flour Composites with Purified Biosustainably Produced Poly(3-Hydroxybutyrate-Co-3-Hydroxyvalerate). Materials.

[38] El Dessouky W.I., Abbas R., Sadik W.A., El Demerdash A.G.M., Hefnawy A. (2017). Improved Adhesion of Superhydrophobic Layer on Metal Surfaces via One Step Spraying Method. Arab. J. Chem..

[39] (2016). ASTM-E96/E96M Standard Test Methods for Water Vapor Transmission of Materials.

[40] Morita K., Gonzales J., Sakaue H. (2018). Effect of PTFE Particle Size on Superhydrophobic Coating for Supercooled Water Prevention. Coatings.

[41] Darmawan A., Utari R., Saputra R.E., Suhartana, Astuti Y. (2018). Synthesis and Characterization of Hydrophobic Silica Thin Layer Derived from Methyltrimethoxysilane (MTMS). IOP Conf. Ser. Mater. Sci. Eng..

[42] Karlina L., Azmiyawati C., Darmawan A. (2019). Synthesis and Characterization of Hydrophobic Silica Prepared by Different Acid Catalysts. IOP Conf. Ser. Mater. Sci. Eng..

[43] Guillaume C., Pinte J., Gontard N., Gastaldi E. (2010). Wheat Gluten-Coated Papers for Bio-Based Food Packaging: Structure, Surface and Transfer Properties. Food Res. Int..

[44] Melendez-Rodriguez B., Castro-Mayorga J.L., Reis M.A.M., Sammon C., Cabedo L., Torres-Giner S., Lagaron J.M. (2018). Preparation and Characterization of Electrospun Food Biopackaging Films of Poly(3-Hydroxybutyrate-Co-3-Hydroxyvalerate) Derived from Fruit Pulp Biowaste. Front. Sustain. Food Syst..

[45] Casasola R., Thomas N.L., Trybala A., Georgiadou S. (2014). Electrospun Poly Lactic Acid (PLA) Fibres: Effect of Different Solvent Systems on Fibre Morphology and Diameter. Polymer.

[46] De Vrieze S., Van Camp T., Nelvig A., Hagström B., Westbroek P., De Clerck K. (2009). The Effect of Temperature and Humidity on Electrospinning. J. Mater. Sci..

[47] Somsap J., Kanjanapongkul K., Tepsorn R. (2018). Effect of Parameters on the Morphology and Fibre Diameters of Edible Electrospun Chitosan-Cellulose Acetate Gelatin Hybrid. MATEC Web Conf..

[48] Cobos C.M., Garzón L., López Martinez J., Fenollar O., Ferrandiz S. (2019). Study of Thermal and Rheological Properties of PLA Loaded with Carbon and Halloysite Nanotubes for Additive Manufacturing. Rapid Prototyp. J..

[49] Wang Y., Chen R., Cai J.Y., Liu Z., Zheng Y., Wang H., Li Q., He N. (2013). Biosynthesis and Thermal Properties of PHBV Produced from Levulinic Acid by Ralstonia Eutropha. PLoS ONE.

[50] Cherpinski A., Szewczyk P.K., Gruszczyński A., Stachewicz U., Lagaron J.M. (2019). Oxygen-Scavenging Multilayered Biopapers Containing Palladium Nanoparticles Obtained by the Electrospinning Coating Technique. Nanomaterials.

[51] Quiles-Carrillo L., Montanes N., Lagaron J.M., Balart R., Torres-Giner S. (2019). In Situ Compatibilization of Biopolymer Ternary Blends by Reactive Extrusion with Low-Functionality Epoxy-Based Styrene–Acrylic Oligomer. J. Polym. Environ..

[52] Long M., Peng S., Chen J., Yang X., Deng W. (2016). A New Replication Method for Fabricating Hierarchical Polymer Surfaces with Robust Superhydrophobicity and Highly Improved Oleophobicity. Colloids Surf. Physicochem. Eng. Asp..

[53] Li Y., Bi J., Wang S., Zhang T., Xu X., Wang H., Cheng S., Zhu B.-W., Tan M. (2018). Bio-Inspired Edible Superhydrophobic Interface for Reducing Residual Liquid Food. J. Agric. Food Chem..

