# Supplementary material for: Super-Repellent Paper Coated with Electrospun Biopolymers and Electrosprayed Silica of Interest in Food Packaging Applications
Source: Nanomaterials (Basel). 2021 Dec 10;11(12):3354. doi: 10.3390/nano11123354 (PMC8706152; doi:10.3390/nano11123354)
Supplement: Supplementary file 1 [file nanomaterials-11-03354-s001.zip › nanomaterials-1479419-supplementary.pdf]

Supplementary Materials

Super-Repellent Paper Coated with Electrospun Biopolymers and Electrosprayed Silica of Interest in Food Packaging Applications

Alvaro Lafraya [1], Cristina Prieto [1], Maria Pardo-Figuerez [1,2], Alberto Chiva [2] and Jose M. Lagaron [1,*]

[1] Novel Materials and Nanotechnology Group, Institute of Agrochemistry and Food Technology (IATA), Spanish Council for Scientific Research (CSIC), Calle Catedrático Agustín Escardino Benlloch 7, 46980 Paterna, Spain; alafraya@iata.csic.es (A.L.); cprieto@iata.csic.es (C.P.); mpardo@iata.csic.es (M.P.-F.)

[2] Bioinicia R&D Department, Bioinicia S.L., Calle Algepser 65, nave 3, 46980 Paterna, Spain; achiva@bioinicia.com

* Correspondence: lagaron@iata.csic.es; Tel.: +34-963-900-022

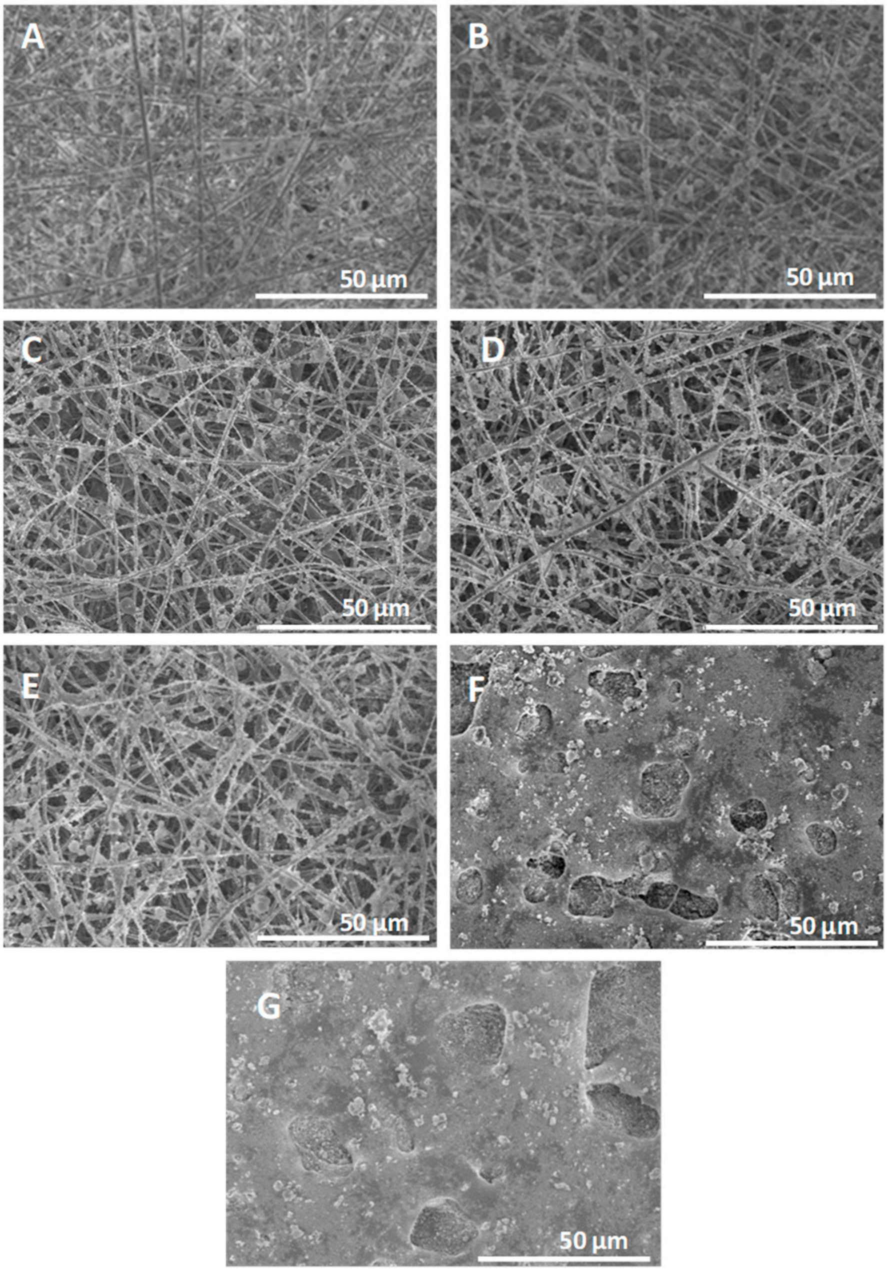

Figure S1. Field emission scanning electron microscope (FE-SEM) images of the multilayer structures based on paper, electrospun polylactide (PLA) and electrosprayed hydrophobic silica microparticles (Paper/PLA/SiO_2) at different annealing temperatures for 20 s. A: No annealing; B: 130 °C; C: 140 °C; D: 150 °C; E: 160 °C; F: 170 °C; G: 180 °C.

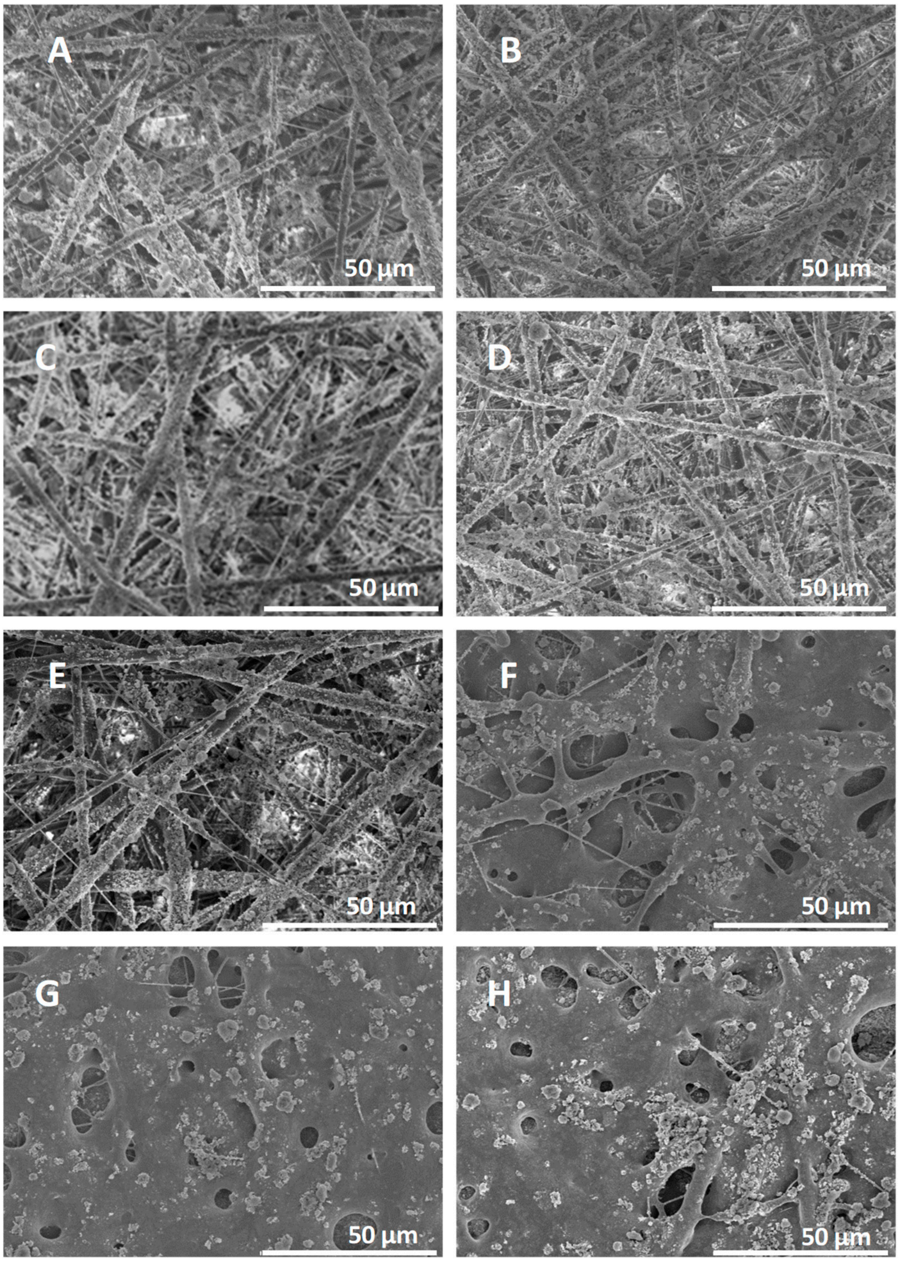

Figure S2. Field emission scanning electron microscope (FE-SEM) images of the multilayer structures based on paper, electrospun poly(ε-caprolactone) (PCL) and electrosprayed hydrophobic silica microparticles (Paper/PCL/SiO_2) at different annealing temperatures for 20 s. A: No annealing; B: 40 °C; C: 45 °C; D: 50 °C; E: 55 °C; F: 60 °C; G: 65 °C; H: 70 °C.

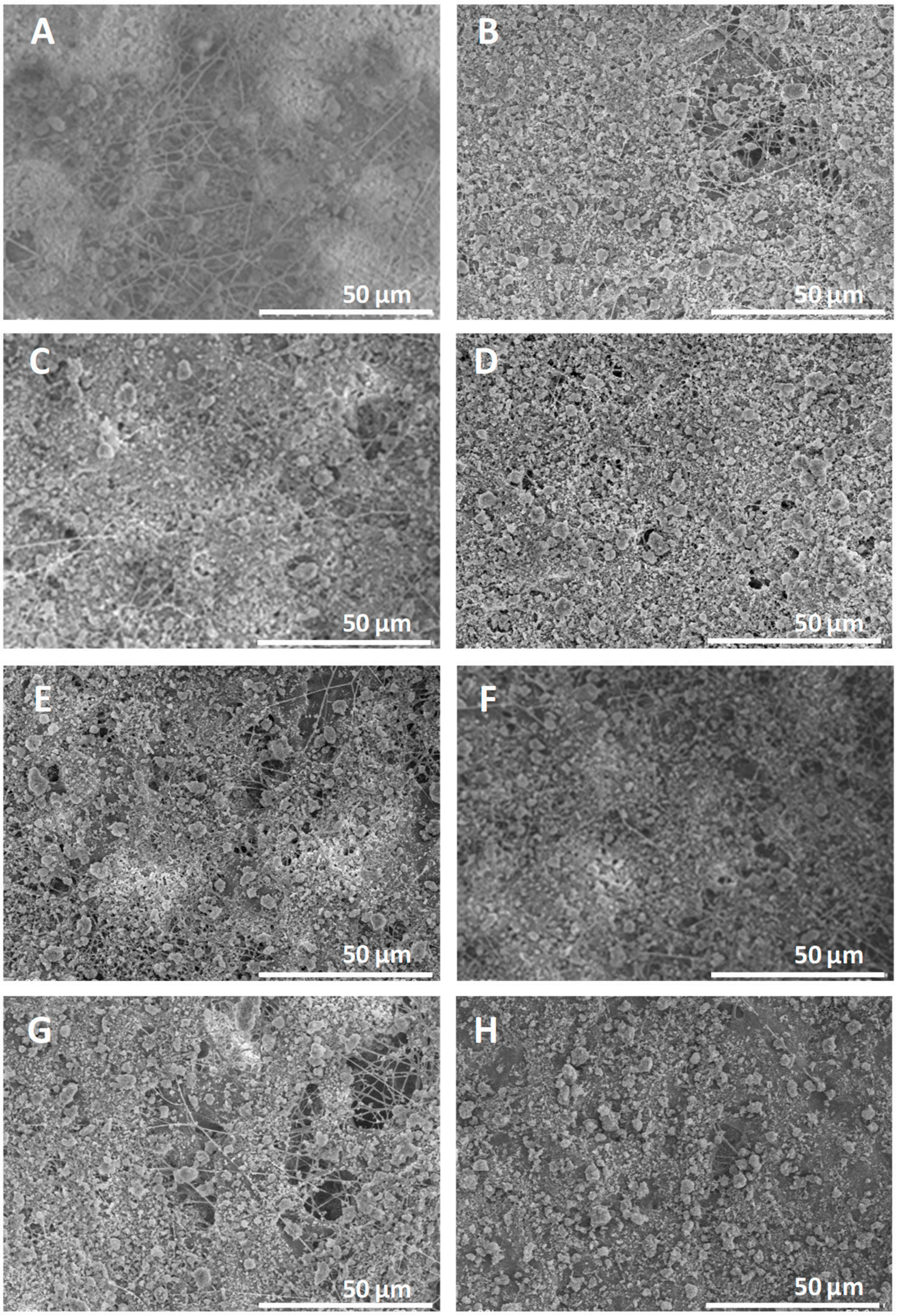

Figure S3. Field emission scanning electron microscope (FE-SEM) images of the multilayer structures based on paper, electrospun poly(3-hydroxybutyrate-co-3-hydroxy-valerate) (PHBV), and electrosprayed hydrophobic silica microparticles (Paper/PHBV/SiO_2) at different annealing temperatures for 20 s: A: No annealing; B: 90 °C; C: 100 °C; D: 110 °C; E: 120 °C; F: 130 °C; G: 140 °C; H: 150 °C.
